# Enhancing Healthcare Providers’ Response to Gender-Based Violence: Development and Validation of an Assessment Tool Using the Modified e-Delphi Approach

**DOI:** 10.7759/cureus.71060

**Published:** 2024-10-08

**Authors:** Namratha Kulkarni, Rizwana Shaikh, Pavan Havaldar

**Affiliations:** 1 Public Health, Jagadguru Gangadhar Mahaswamigalu Moorsavirmath Medical College, Karnataka Lingayat Education (KLE) Academy of Higher Education and Research, Hubballi, IND; 2 Anatomy, Gadag Institute of Medical Sciences, Gadag, IND

**Keywords:** attitudes, gender-based violence, healthcare providers, india, knowledge, modified delphi method, practices, questionnaire validation

## Abstract

Background

Effective gender-based violence (GBV) interventions depend on precise tools to assess healthcare providers’ knowledge, attitudes, and practices (KAP). This study aimed to develop and validate a culturally appropriate GBV questionnaire on the KAP of healthcare workers in India using a modified e-Delphi method.

Methodology

The study used a modified e-Delphi approach, conducted online, to validate a GBV questionnaire. The process included the following three rounds: content validation, quantitative validation, and finalization. It began with a literature review and questionnaire drafting in May 2022, followed by validation from September 2022 to December 2023. The expert panel included healthcare professionals, public health experts, and researchers. The questionnaire included the following four sections: demographic information, knowledge of GBV, attitudes toward GBV, and GBV-related practices.

Results

In Round 1, significant revisions were made to enhance relevance and clarity, including the addition of a “Don’t Know” option and revisions to questions on gender roles and societal expectations. The quantitative validation of Round 2 revealed high scores for Importance (4.980), Usefulness (4.965), Relevance (4.965), Clarity (4.955), Simplicity (4.980), and Ambiguity (4.815), with all items retained and no significant modifications. In Round 3, consensus was achieved, finalizing the questionnaire with expert validation certificates, demonstrating strong reliability and contextual appropriateness.

Conclusions

The validated KAP questionnaire is a reliable tool for assessing healthcare providers’ capacity to manage GBV. It can be used to inform and enhance interventions aimed at improving GBV management in healthcare settings.

## Introduction

Gender-based violence (GBV) is a serious issue rooted in social, cultural, and economic inequalities, especially in patriarchal societies like India. GBV appears in various forms, i.e., physical, sexual, emotional, and economic, affecting women across all demographics. These acts of violence are human rights violations with severe medical, psychological, and social impacts. Women experiencing GBV face numerous health issues, including injuries, reproductive health problems, mental disorders, and chronic conditions, which deeply affect their quality of life [1-4].

Healthcare providers, often the first contact for GBV victims, play a crucial role in identification, management, and support. However, their ability to effectively address GBV is often limited by a lack of knowledge, inadequate training, and entrenched social biases. Many healthcare workers struggle to recognize signs of GBV or hesitate to intervene due to cultural norms. This highlights the need for targeted training and capacity-building to equip healthcare providers with the skills and knowledge required to address GBV effectively [4,5].

The authors are currently conducting a funded parent study titled “Catalyzing Change: Enhancing the Capacity of Healthcare Workers to Address Violence Against Women,” which aims to validate and utilize a questionnaire to assess healthcare providers’ knowledge, attitudes, and practices (KAP) concerning GBV. Additionally, the study seeks to build the capacity of these healthcare workers to take effective action against GBV. The research is taking place in the field practice area of Karnataka Lingayat Education’s Jagadguru Gangadhar Mahaswamigalu Moorsavirmath Medical College, Karnataka, and is organized into the following two cohorts: Cohort I includes accredited social health activists and Anganwadi workers, while Cohort II comprises primary and urban health center staff, such as auxiliary nurse midwives, nurses, medical officers, and support staff. One of the objectives of this study is to develop and refine a culturally appropriate questionnaire tailored to assess the KAP of healthcare providers regarding GBV in India. The research also focuses on ensuring the reliability and validity of this instrument to effectively identify gaps in KAP and guide targeted interventions to improve healthcare workers’ ability to address GBV.

The questionnaire validation in the parent study is crucial due to the lack of widely accepted tools tailored to assess healthcare providers’ KAP regarding GBV in India. Existing instruments often miss the unique challenges and cultural nuances, particularly in community health settings. As these providers are often the first contact for women experiencing violence, it is essential that the assessment tools are context-specific and comprehensive to accurately measure their readiness and capacity [5].

To address this gap, we used the modified e-Delphi method for questionnaire validation, allowing for iterative refinement based on expert input on the complexities of GBV in India. This ensured the final tool is both reliable and valid, while grounded in the practical realities healthcare providers face in managing GBV cases. Developing a validated, context-specific questionnaire is crucial for enhancing the effectiveness of training and intervention programs, as it reflects local needs and helps identify knowledge and practice gaps, guiding targeted capacity-building efforts to better combat violence against women in India [6,7].

## Materials and methods

Study design

**Figure 1:**
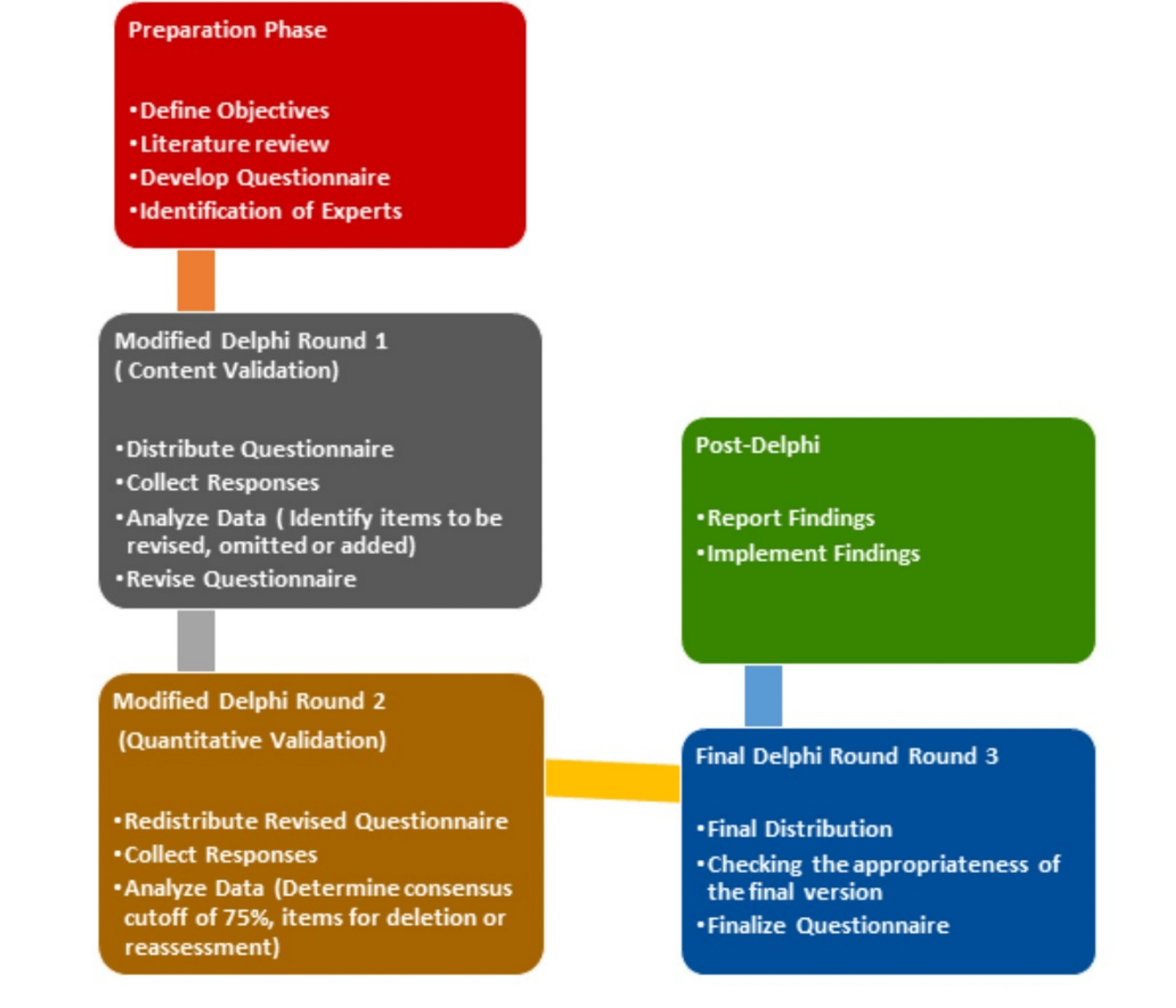
Steps in the modified e-Delphi method of validation of the questionnaire.

The questionnaire was validated using a modified e-Delphi method, conducted entirely via email and online platforms, removing the need for physical meetings. This method provided a rigorous and adaptable framework for ensuring the questionnaire’s contextual appropriateness and methodological soundness in evaluating GBV-related KAP among healthcare providers. The process began with a literature review and initial drafting of the questionnaire in May 2022, followed by the validation process from September 2022 to December 2023. Figure 1 outlines the key steps, right from the preparation phase with objective setting, literature review, questionnaire development, and expert identification, followed by the following three rounds: content validation, quantitative validation, and finalization, culminating in the reporting and implementation of findings in the modified e-Delphi method used for questionnaire validation [8,9].

Study questionnaire

The questionnaire is organized into four main sections designed to gather comprehensive data on participants’ KAP and preparedness regarding GBV. Section I collects demographic information, including details such as name, age, sex, department, designation, years of experience, address, religion, educational qualification, and marital status. Section II is divided into three parts. Part A includes 18 statements that assess participants’ knowledge of GBV, intimate partner violence (IPV), and related issues, with responses in “Yes,” “No,” or “Don’t Know” format. Part B contains 10 statements evaluating attitudes toward GBV, including views on violence against women, gender roles, and IPV, measured on a five-point Likert scale ranging from “Strongly Disagree” to “Strongly Agree.” Part C focuses on assessing the frequency of practices related to identifying and addressing GBV through 12 statements, with responses indicating the frequency of actions on a scale from “Never Done” to “Always Do.” Finally, Section III includes fill-in-the-blank questions for specific data, such as the central women’s helpline number and the number of GBV cases identified and reported over the past year. This structured approach ensures a thorough understanding of participants’ KAP concerning GBV, facilitating targeted interventions and improvements in support services [1,5,10-15].

Modified e-Delphi panel

The expert panel for this study was carefully selected based on their interest and involvement in public health. Recruitment started with an email invitation containing detailed study information and consent forms, followed by phone calls to address any questions and provide clarification. Selection criteria included healthcare professionals registered with the Indian Medical Register or the Karnataka Medical Council, and a willingness to participate online and access email. This diverse composition ensured a comprehensive and impartial reflection of current knowledge and perceptions related to health and social issues. Participants were excluded from the expert panel if they did not respond to the email invitation even after two telephonic reminders or did not consent to participate. Anonymity among panel members promoted unbiased feedback, and their combined expertise was essential for the rigorous validation of the questionnaire.

The panel consisted of five experts: two from national-level non-governmental organizations specializing in public health and sociology, already working on GBV projects, focusing on documentation, data quality, and monitoring; one expert in research methodology; one expert in research methodology and statistics; and one with a background in ethics and research.

Round 1

The pre-validation questionnaire had four sections and 55 items. Section I included 11 demographic questions. Section II had 24 items split into two parts. Part A covered 13 items on knowledge of gender roles, violence, and IPV, while Part B focused on attitudes toward GBV with seven items. Section III evaluated practices with nine items about IPV preparedness. Section IV contained four fill-in-the-blank questions on helplines and reporting.

In Round 1, the initial draft of the questionnaire was reviewed by experts who provided feedback on its relevance, clarity, and comprehensiveness. The experts suggested additions and deletions of questions, modifications in language, and recommended questions based on the content required to assess healthcare providers’ KAP regarding GBV. Their input ensured the questionnaire effectively captured key constructs and addressed the specific needs of the Indian context. Revisions based on their feedback refined the instrument to align with the study’s objectives and practical application [8,9].

Round 2

In the second round of validation, the revised questionnaire was evaluated quantitatively by experts using a five-point Likert scale, as highlighted in Table 1. Each item was assessed based on the following six criteria: Importance (the significance of the question to the overall purpose), Usefulness (how effectively the question contributes valuable information), Relevance (the question’s pertinence to the study topic), Clarity (how the question is formulated and understood), Simplicity (whether the question is straightforward to answer), and Ambiguity (potential for confusion or misinterpretation).

**Table 1:** Likert scale criteria for each item.

Criteria	1	2	3	4	5
Importance	Not at all important	Slightly important	Relatively important	Important	Very important
Relevance	Not relevant	Item needs revision	Relevant needs a minor change	Relevant	Very relevant
Clarity	Unclear	Item needs revision	Clear needs a minor change	Clear	Very clear
Simplicity	Not simple	Item needs revision	Simple needs a minor change	Simple	Very simple
Ambiguity	Doubtful	Item needs revision	Not ambiguous needs a minor change	Not ambiguous	Meaning is clear
Usefulness	Unnecessary and not useful	Useful but not necessary	Useful and necessary to some extent	Useful and necessary	Very useful and necessary

Additionally, experts assessed the questionnaire’s feasibility and length. Feasibility was rated from “Very Good” to “Very Poor,” while length was evaluated from “Too Short” to “Too Long.” This detailed evaluation ensured that the questionnaire was practical and aligned with the study’s objectives [8,16-20].

Consensus

Consensus for validating a question is achieved if at least 75% of the expert panel rates it 4 or above. For a panel of five experts, this means at least four (80%) experts must give a score of 4 or higher. Consensus is determined by tallying the number of experts rating each question 4 or above. If this number meets or exceeds the 75% threshold, the question is deemed to have achieved consensus.

Consensus was determined by calculating the mean Likert score for each criterion based on ratings from all reviewers. The mean of these mean Likert scores across all criteria is then computed. If this mean of mean Likert score is 4 or above, and at least 75% of the experts have rated the item with a score of 4 or higher, the question is considered to have met the consensus and is retained. Items with a mean of mean Likert score between 2 and 3 are considered for modification, while those with a score below 2 are deleted. To analyze the importance of each question, the total score from all reviewers was summed and divided by the number of reviewers to compute the mean Likert score for importance. This process was repeated for all criteria, i.e., Usefulness, Relevance, Clarity, Simplicity, and Ambiguity. The average of these mean scores across the six criteria was then used to determine the overall consensus [8,16-20].

Round 3 and post-e-Delphi

After achieving consensus in the third round, the questionnaire was finalized and validated, and panelists provided a certificate of validation. The validated questionnaire was then prepared for distribution to the target population. Detailed instructions were developed to guide participants and ensure consistent data collection. This marked the transition from validation to application, with the tool now set to gather insights and inform strategies for improving GBV-related training and policies in healthcare [16-20].

## Results

Round 1

The qualitative and content validation of the questionnaire for “Catalyzing Change: Enhancing the Capacity of Healthcare Workers to Address Violence Against Women” led to several revisions. The process resulted in adding new items, removing irrelevant questions, and modifying existing ones for clarity and relevance. A “Don’t Know” option was introduced, and new questions explored gender roles and societal expectations, such as beliefs about gender-specific interests and career pursuits. Definitions of IPV and domestic violence were expanded, and explanations of moral violence were included. The questionnaire was also translated into the local language Kannada for better accessibility, and questions discussing abuse with IPV victims were revised. Table 2 summarizes the content validation, highlighting the actions taken in the questionnaire’s knowledge and attitudes sections.

**Table 2:** Summary of content validation.

Section	Action	Details	Justification
Part A: Knowledge	Addition	Nine new questions were added regarding gender roles and societal expectations. Examples: gender-specific interests, non-traditional career choices, and gender pay gaps	Enhances understanding of practical implications of gender roles
Part A: Knowledge	Addition	The “Don’t Know” option was added to capture uncertainty	Provides a more nuanced measure of knowledge
Part A: Knowledge	Deletion	Question on intercaste marriages removed	Did not align with the core focus on GBV
Part A: Knowledge	Modification	Definitions of sex and gender were revised to focus on practical applications related to gender roles	Improves relevance to real-world contexts
Part A: Knowledge	Modification	Clarified domestic violence to include violence by family members beyond the spouse	Ensures comprehensive coverage of domestic violence
Part A: Knowledge	Modification	The moral violence concept was simplified; added examples and translated into local languages	Aims to enhance understanding among field workers
Part B: Attitudes	Modification	Rephrased question on spousal violence to include “in any circumstance”	Increases complexity and better captures attitudes
Part B: Attitudes	Deletion	Question on intercaste marriages removed	Did not fit with the flow of the questionnaire and did not focus on GBV
Part B: Attitudes	Review	To ensure alignment with revised knowledge and attitudes sections	Ensures consistency across the questionnaire sections

Round 2

In Round 2, the questionnaire was evaluated using a five-point Likert scale, resulting in the following mean scores: Importance, 4.980; Usefulness, 4.965; Relevance, 4.965; Clarity, 4.955; Simplicity, 4.980; and Ambiguity, 4.815. Of 111 assessed items, scores ranged from a low of 3.60 to a high of 5.00. The overall mean score was 4.83, with a standard deviation of 0.22 and a variance of 0.048. The skewness was -3.44, with a standard error of skewness of 0.229. Notably, 23.4% of respondents rated items at the maximum score of 5.00, and 18.0% rated items at 4.90.

Updated consensus criteria results

Table 3 summarizes the mean Likert scores and feedback from the validation process.

**Table 3:** Summary of mean Likert scores for each category of items.

Category	Items	Mean Likert score
General information	Name, Age, Sex, Department, Designation, Years of experience, Address, Religion, Educational qualification, Marital status, Age at marriage	4 to 5
Gender roles and stereotypes	Questions about gender-specific interests, career choices, and household responsibilities	Consistently 5
Intimate partner violence (IPV)	Definition and forms of IPV, including spousal violence, moral violence, and reasons for staying in violent relationships	Consistently 5
Identification and response to IPV	Appropriate ways to ask about IPV, reasons for staying in violent relationships, and identifying symptoms	Consistently 5
Skills and resources	Asking about IPV, referring clients, awareness of legal requirements, and documentation	Consistently 5
Attitudes and practices	Views on violence, gender roles, and responses to IPV	Consistently 5
Frequency and actions	Frequency of documentation, notification, safety planning, and contacting support services. Some items related to safety planning have slightly lower scores	Consistently 5

Items to Be Retained

In the second round of quantitative validation, several items were retained without modification. Items, where 75% or more experts rated them with a score of 4 or above, were kept as they demonstrated strong consensus on their quality and relevance. Examples of such items include those related to basic demographic information such as age, sex, and educational qualification, all of which received a mean Likert score of 5. Questions regarding domestic violence definitions and related aspects, as well as those on IPV, referral, and support services, also received high scores, typically around 5. Additional items retained without modification included those assessing the destruction of objects, moral violence variants (such as detention and withholding of rights), and aspects of danger and safety plans, all of which had mean scores of 4.

Items to Be Deleted

No items were identified for deletion.

Items to Be Modified

Items that received mostly ratings of 2 or 3, indicating a mean Likert score of less than 4, were reviewed; however, no items were identified with scores of 2 or 3.

Feasibility of administering the questionnaire

The questionnaire received high ratings for feasibility, with four out of five experts rating it as “Very Good” (scored as 5) and one expert rating it as “Good” (scored as 4). The mean feasibility score was 4.8.

Length of the questionnaire

The questionnaire was assessed for its length, with three out of five experts rating it as “Long” (scored as 4) and two experts rating it as “Too Long” (scored as 5). The mean length score was 4.4.

## Discussion

In the first round of the modified e-Delphi method, the questionnaire underwent significant revisions to enhance its relevance and clarity. New questions were introduced to address gender roles and societal expectations, while a “Don’t Know” option was added to capture more nuanced responses. Irrelevant questions, such as those related to intercaste marriages, were removed, and complex concepts such as “moral violence” were simplified and translated into local languages to improve comprehension. Additionally, the question on spousal violence was rephrased for accuracy, and the Attitudes section was updated to align with the revised Knowledge section, ensuring consistency across the questionnaire. This approach is similar to the methodology in developing the Global Sexual Health Survey [19], which involved iterative feedback and refinement through crowdsourcing and the Delphi method.

In the second round, quantitative validation scores reflected the questionnaire’s robustness. High scores for Importance and Simplicity (both at 4.980) indicated its relevance and ease of understanding. Usefulness and Relevance were also rated highly at 4.965, demonstrating the questionnaire’s practical value in assessing key areas. Clarity, though slightly lower compared to other aspects at 4.955, suggested only minor concerns regarding the precision of some questions. The lowest score was for Ambiguity, which, while still high at 4.815, indicated that certain questions required further refinement. These results are consistent with findings from the Questionnaire of Intention to Help in VAW Cases (QIHVC) [17], where similar multi-phase validation, including Delphi methods, emphasized the importance of iterative feedback in refining survey instruments. The overall mean score of 4.83, with low variability (standard deviation of 0.22, variance of 0.048), demonstrated consistency and reliability in the feedback. The negative skewness of -3.44 further supported strong agreement among respondents, reinforcing the questionnaire’s reliability, as seen in the development of the Male Partners’ Attendance at Childbirth-Questionnaires (MPAC-QHMUs and MPAC-QMS) [8], where consistent expert feedback led to the refinement of tools for different stakeholder groups.

After the final round, when the consensus was achieved, the expert panel provided certificates of validation, formally endorsing the questionnaire. This endorsement confirmed the tool’s methodological soundness and contextual appropriateness for assessing GBV-related KAP among healthcare providers. The feasibility of administering the questionnaire was rated highly, with a mean score of 4.8, indicating its practical suitability. However, concerns about the questionnaire’s length, with a mean score of 4.4, suggested that streamlining could improve respondent engagement without sacrificing content comprehensiveness. This mirrors the process seen in other studies, where validation and feedback loops were crucial for developing robust and reliable instruments that are both practical and comprehensive.

**Figure 2:**
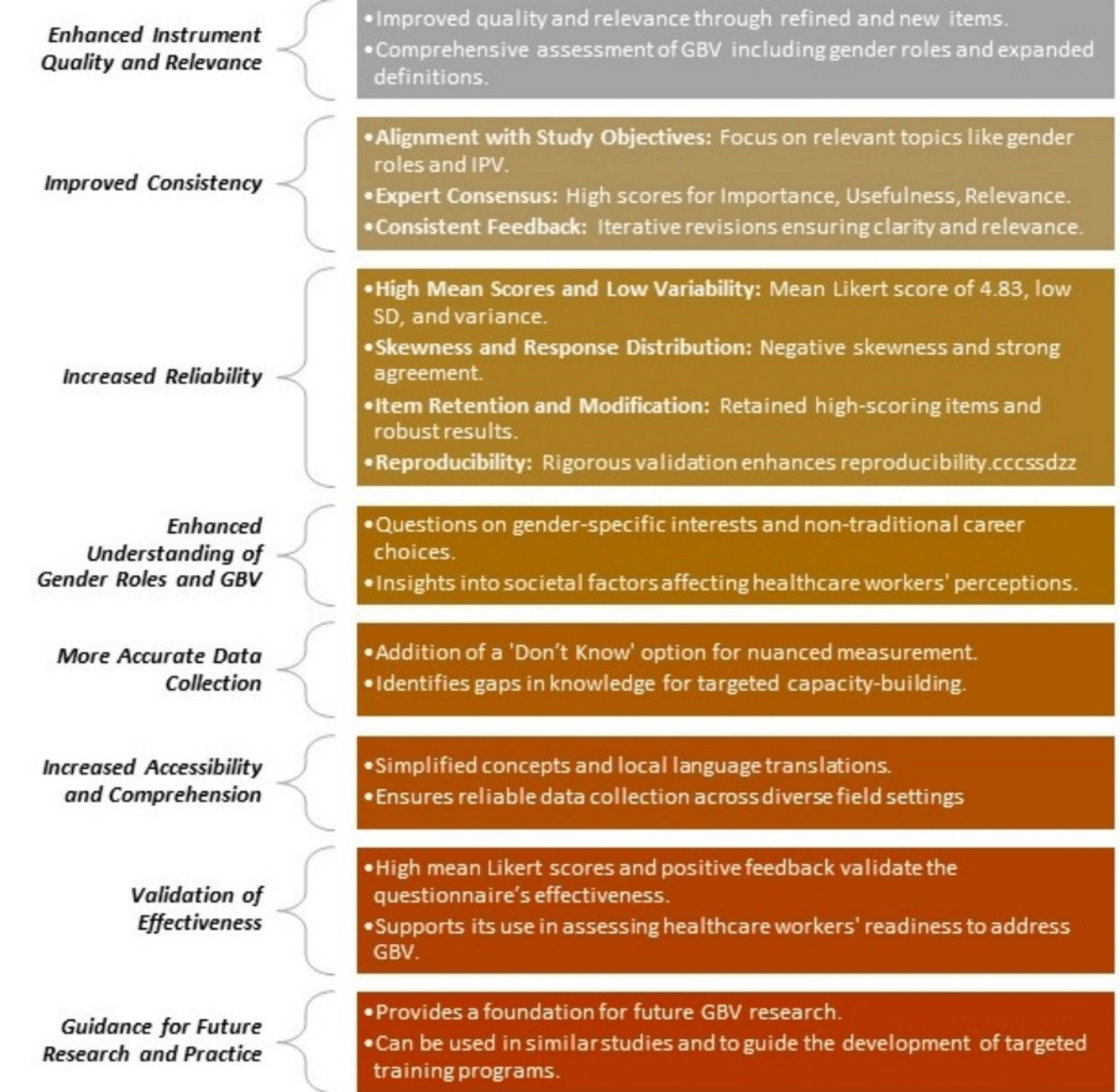
Implications and outcomes of the GBV questionnaire validated by the modified e-Delphi method. IPV: intimate partner violence; GBV: gender-based violence

The validated questionnaire now serves as a reliable tool for assessing GBV-related KAP among healthcare providers, offering a strong foundation for targeted interventions and policy development in healthcare settings. Figure 2 [20,21] illustrates the implications and outcomes of the GBV questionnaire validated by the modified e-Delphi method, highlighting enhanced instrument quality, increased reliability, improved consistency, and more accurate data collection. The validation process also led to a deeper understanding of gender roles and GBV and increased accessibility and comprehension through simplified concepts and local language translations. These outcomes not only validate the effectiveness of the questionnaire but also provide a foundation for future research and practice, supporting its use in similar studies and guiding the development of targeted training programs.

This study has several limitations common to validation research. The small expert panel might limit perspective breadth; however, a balance is crucial to avoid over-representation. The questionnaire, designed specifically for the Indian context, may need adaptation for other settings. The Likert scale could have led to overly favorable ratings. It lacks assessment of other reliability forms, such as test-retest reliability. Further real-world testing is needed to confirm reliability and validity. Additionally, self-reported data may introduce response bias, though anonymity and confidentiality can help mitigate this.

## Conclusions

The modified e-Delphi method effectively refined the questionnaire assessing healthcare providers’ KAP regarding GBV through expert feedback, ensuring its reliability and relevance. The validated questionnaire provides a robust framework for identifying gaps, guiding training programs, and advancing future GBV research in India. Further research is recommended to test the tool’s effectiveness in broader settings.

## Appendices

Annexure 1

As the questionnaire is currently undergoing the copyright process, we cannot disclose or publish the exact details publicly until the process is complete. Below is a modified version that illustrates the content and structure of the questionnaire.

**Table 4:** A concise summary of the various sections, topics, and key points in the questionnaire, along with the type of response format for each part.

Part	Section	Topics	Examples of statements/questions	Response format	Cohort
	Demographic details	Participant information	Name, Age, Sex, Designation, Experience, Education, Marital status		I and II
A	Knowledge	Gender roles & expectations	- Do you believe that society still expects boys and girls to have distinct interests and toys? - Do people think boys should only like “boy” things, and girls “girl” things? - Should boys be encouraged to express emotions? - Can anyone, regardless of gender, be a nurse, engineer, or firefighter?	Yes/No/Don’t Know	I and II
		Intimate partner violence (IPV) & domestic violence (DV)	- IPV is typically violence between intimate partners. - In India, IPV is referred to as spousal violence and is included under DV. - Forced sexual intercourse by an intimate partner is a form of gender violence	Yes/No/Don’t Know	I and II
		Types of violence	- Defaming, hurting, humiliating, and intimidating women are forms of violence by intimate partners. - Detention and withholding rights are forms of moral violence	Yes/No/Don’t Know	I and II
		Reasons for aggression	- Habitual wife beating is normal in some families. - Unemployment and alcohol/drug abuse lead to violence	Yes/No/Don’t Know	I and II
		Barriers to leaving violent relationships	- Financial dependence on the perpetrator. - Religious beliefs and fear of isolation	Yes/No/Don’t Know	I and II
		Warning signs of abuse	- Frequent injuries, repeated unwanted pregnancies, chronic pain, psychological problems, or multiple health consultations without a clear diagnosis	Yes/No/Don’t Know	I and II
		Approaches to asking about IPV	- Are you a victim of domestic violence? - Are you afraid of your partner?	Yes/No/Don’t Know	II
		Knowledge & skills	- Knowledge of how to discuss abuse with female/male victims or those from different cultures. - Awareness of legal requirements for reporting GBV	Yes/No/Don’t Know	II
B	Attitudes	Attitudes toward GBV	- It is right for a man to beat his wife in any circumstance. - Husband’s and in-laws’ approval is essential for a woman to use family planning methods. - Aggression towards a woman by her husband is a private matter	Strongly Disagree to Strongly Agree	I and II
		Gender roles	- Cooking is a woman’s responsibility. - Earning for the family is the man’s responsibility. - Taking care of children and the elderly is a woman’s responsibility	Strongly Disagree to Strongly Agree	I and II
		Acceptability of violence	- It is acceptable for a man to hit his wife if she fails to perform domestic duties or if he suspects she is unfaithful	Strongly Disagree to Strongly Agree	I and II
		Attitudes toward asking about violence	- Asking about domestic violence invades privacy or is humiliating. - Healthcare providers do not have time to assist with IPV	Strongly Disagree to Strongly Agree	II
		Views on victims of abuse	- If a victim stays in a violent relationship, they must accept responsibility. - Victims have the right to decide if hospital staff should intervene	Strongly Disagree to Strongly Agree	II
C	Practices & preparedness	IPV identification & support	- I ask all new families in my area about abuse in their relationships. - I refer women to support services (medical, psychological, legal, etc.). - I assess the immediate danger level after sexual assault.	Never Done to Always Do	I and II
		Documentation & referral	- I document patient statements and physical findings in the chart. - I notify authorities when mandated and provide referrals.	Never Done to Always Do	II
D	Miscellaneous	GBV Helpline & case reports	- The central toll-free women’s helpline number is _____. - Number of GBV cases identified and reported from ---- to -----	Fill in the Blanks	I and II
